# Workers’ characteristics associated with the type of healthcare provider first seen for occupational back pain

**DOI:** 10.1186/s12891-016-1298-y

**Published:** 2016-10-18

**Authors:** Marc-André Blanchette, Michèle Rivard, Clermont E. Dionne, Sheilah Hogg-Johnson, Ivan Steenstra

**Affiliations:** 1 Public Health PhD Program, School of Public Health, University of Montreal, Montreal, QC Canada; 2 Department of Social and Preventive Medicine, School of Public Health, University of Montreal, Montreal, QC Canada; 3 Public Health Research Institute, University of Montreal, Montreal, QC Canada; 4 Department of Rehabilitation, Faculty of Medicine, Université Laval, Québec City, QC Canada; 5 Axe Santé des populations et pratiques optimales en santé, Centre de recherche du CHU de Québec (CHUQ), Québec City, QC Canada; 6 Institute for Work & Health, Toronto, ON Canada; 7 Dalla Lana School of Public Health, University of Toronto, Toronto, ON Canada

**Keywords:** Back pain, Primary care, Healthcare provider, Medical doctor, Chiropractic, Physiotherapy, Determinants, Occupational health

## Abstract

**Background:**

Few studies have compared the factors that drive patients’ decision to choose a chiropractor, physician or physiotherapist as their first healthcare provider for occupational back pain. The purpose of this study is to identify characteristics associated with the choice of first healthcare provider seen for acute uncomplicated occupational back pain.

**Methods:**

We analyzed data collected by the Workplace Safety and Insurance Board from a cohort of workers with compensated back pain in 2005 in Ontario (Canada). Multivariable logistic regression models were created to identify factors associated with the type of first healthcare provider seen (chiropractor, physician, or physiotherapist). Adjustments to the final models were evaluated using the area under the receiver-operating characteristics curve (ROC).

**Results:**

According to the 5520 analyzed claims, 85.3 % of the patients saw a physician, 11.4 % saw a chiropractor, and 3.2 % saw a physiotherapist. Longer job tenure (odds ratio (OR) = 1.02, *P* = 0.004), higher gross personal income (*OR* = 1.06, *P* = 0.018), mixed-manual job (*OR* = 1.35, *P* = 0.004) and previous similar injury (*OR* = 1.60, *P* < 0.001) increased the odds of seeing a chiropractor rather than a physician, while the size of the community (>500,000 inhabitants) and the availability of an early return to work program in the workplace (*OR* = 0.77, *P* = 0.035) decreased it. The odds of seeing a physiotherapist rather than a physician increased with increasing age (*OR* = 1.19, *P* = 0.019), previous similar injury (*OR* = 1.71, *P* < 0.001) and severity of injury (*OR* = 2.03, *P* = 0.010). Increased age (*OR* = 1.28, *P* = 0.008) and size of community (>1,500,000 inhabitants; *OR* = 2.58, *P* = 0.002) increased the odds of seeing a physiotherapist rather than a chiropractor, while holding a mixed-manual job significantly decreased those odds (*OR* = 0.63, *P* = 0.044). The area under the ROC curve of our multivariable models varied from 0.62 to 0.64.

**Conclusion:**

The type of first healthcare provider sought for occupational back pain is influenced by injury-and work-related factors and by the worker’s age, income and community size. Contrary to previous studies, the workers who first sought a physician did not have higher odds of having a severe injury.

**Electronic supplementary material:**

The online version of this article (doi:10.1186/s12891-016-1298-y) contains supplementary material, which is available to authorized users.

## Background

Among the general population, low back pain is a common non-fatal condition with a point prevalence of 9.4 % [1] and a lifetime prevalence of approximately 85 % [2, 3]. It was estimated that about one-third of low back pain is attributed to occupation [4, 5] and that occupational low back pain represents one-third of all disability related to occupational factors considered in the Global Burden of Disease study [5]. Although the majority of patients with back pain show significant improvement within the first month [6], symptoms are often recurrent or chronic [7]. As a result, back pain is a leading cause of disability worldwide [8], and it ranks sixth among the health problems that generate the most direct medical costs in North America [9]. In Ontario (Canada), low back pain in the most common occupational injury compensated by the Workplace Safety and Insurance Board (WSIB) even if the percentage of all lost time claims attributable to low back pain decreased from 19.9 % (*n* = 11,290) to 16.8 % (*n* = 8,677) between 2011 and 2015 [10]. It is considered a type of claim that has as high an impact on workers and employers as shoulder and fracture claims. These three claim types are responsible for more than 40 % of all benefits payments [11].

In Ontario, the WSIB plays the role of public insurer to workers and employers. It provides financial support, medical assistance and rehabilitation for return to work. On January 1, 2004, as part of the commitment to quality and timely healthcare, the WSIB revised its policy on Choice and Change of Health Professional (17-01-03) in the Operational Policy Manual. Previously, workers could go directly (without referrals from other professionals) to a physician or a chiropractor; the new policy expanded direct access to physiotherapists and registered nurses (extended class). The new policy was particularly innovative because physiotherapists have traditionally been used in specialized care (after a medical referral) in Canada and the United States [12, 13]. According to a systematic review of studies conducted between 1993 and 2012, direct access to physiotherapy care was associated with better patient outcomes and satisfaction and a reduction in the use of healthcare resources (number of consultations, imaging, medication, consultations with other providers) and costs [14].

A previous meta-analysis of population-based studies showed that among the general population, female gender, previous history of back pain, pain intensity and a high level of disability are associated with an increased probability of seeking care for low back pain [15]. Factors associated with the type of care sought have been studied among the general population [16], among people with back pain [12, 13, 17–33] and among injured workers [33, 34]. Most of the studies originate from the United-States [12, 16, 17, 20, 22, 24, 26, 28, 30–34], but some are from Australia [27, 35], Canada [13, 16, 25, 33], Denmark [18], France [23], Israel [29], the Netherlands [21], Sweden [36] and Switzerland [19]. Most studies compared chiropractic to medical care [13, 16, 18–20, 24, 26–28, 30, 31, 33, 35, 36], while a few studies included physiotherapy (referral or exclusive care) [12, 16, 21–23, 25, 29, 37], and only one American study specifically compared the three type of professionals as primary healthcare providers [17]. Some of the findings from these studies diverge, suggesting that they might be specific to their jurisdictions [16]. To our knowledge, no study has compared the factors that drive patients’ decision to choose a chiropractor, physician or physiotherapist as their first healthcare provider for occupational back pain.

Understanding the factors that influence the initial pattern of care-seeking for occupational back pain informs researchers about the characteristics of the subpopulations that seek different types of healthcare providers. Recent publications provide limited conclusions and outline the importance of improving knowledge about care-seeking behavior [38–40]. The objective of this study was to identify individual characteristics associated with the choice of first healthcare provider (chiropractor, physician, physiotherapist) sought by workers with occupational low back pain.

## Methods

### Study population

**Fig. 1:**
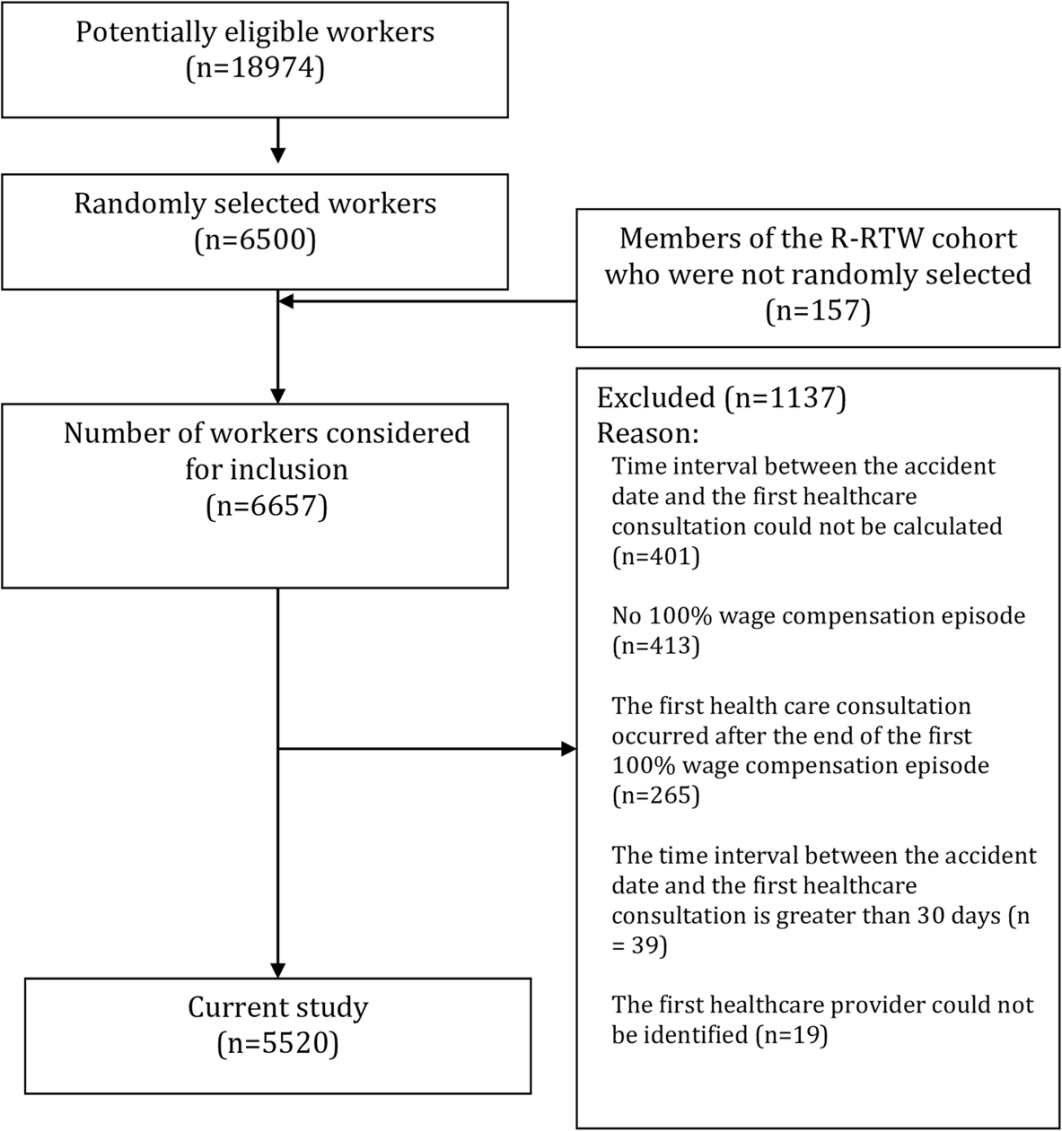
Diagram showing the selection of the study subjects

The study population is a cohort of workers who had filed a lost-time claim with the WSIB for uncomplicated back pain with a date of accident between January 1 and June 30, 2005, based on historical WSIB records. The data related to this study were initially extracted for a project that aimed to predict the time spent receiving benefits [41, 42]. The time period was selected to mimic the recruitment period for the Readiness for Return to Work (R-RTW) cohort [42, 43] and to allow a complete 2 year follow-up of all workers at the time of data assembly. From all of the 18,974 lost-time claims with the part of body and nature of injury related to back pain (Additional file 1: Table S1) and an eligible accident date, a random sample of 6,500 was selected. One hundred fifty-seven back pain subjects from the R-RTW cohort who were not randomly selected were added afterwards because the project for which the data were extracted [41, 42] initially wanted to use the R-RTW cohort as a subgroup of workers with additional information. We excluded workers without any 100 % wage compensation episode and those who had missing or aberrant data regarding our main dependent variable (first healthcare provider). Workers with a long time interval (more than 30 days) before the first recorded health care consultation were excluded because they may be more likely to have sought care outside the compensation system and/or to have backdated the accident date. Our final sample included 5520 injured workers (Fig. 1).

### Data sources

A research agreement was concluded in order to access WSIB data for research purposes. We used data routinely collected by the WSIB from three sources: the WSIB information management catalog (including the claim file); the electronic healthcare billing database; and the database of imaged forms completed by the employers, workers and healthcare providers. The relevant information contained in these databases was extracted and merged by an experienced programmer-analyst at the Institute for Work and Health (IWH).

When a claim for wage compensation is submitted to the WSIB, the worker, the employer and the healthcare provider must complete a number of forms. The employer’s form must be completed within 3 days of the work accident. A delayed or incomplete declaration can lead to a fine. If too much information is missing from the form, the claim cannot be processed; therefore, response rates are high, and the amount of missing data is low. Two data extractors independently accessed the first 100 cases’ forms using WSIB’s imaged files. Because they had a 98 % agreement, only one extractor completed the remaining cases [41]. When information was present on both the employer’s and the worker’s form, we always gave priority to the worker’s version.

The majority of the healthcare providers completed the version of their form that was introduced in 2003. However, some providers used the version that was introduced in 1999, probably because they had an old paper copy of that form in their office. Both versions contained information about task limitations and specifically asked about the worker’s abilities to use public transportation and to operate a motor vehicle. The 1999 version added an open question about any other restrictions that the patient should observe. The 2003 version asked about ten specific task limitations and provided space to list non-prespecified limitations. To include the information from both versions of the healthcare provider form, we considered that the worker to have a task limitation if any type of restriction (prespecified or not) was indicated.

## Variables

### Dependent variable

#### First healthcare provider type

We considered two sources of information to identify the first healthcare provider: the healthcare billing database and the healthcare provider. All the billing “dates of service” prior to the accident date (*n* = 283) were screened for obvious data entry mistakes (e.g., day-month inversion) that would bring the service date very close to the accident date. By considering the billing history, a decision was made to either correct the obvious mistake or to erase the date to select a more credible first date of service from the billing database. A similar process was independently conducted for the first date on the healthcare provider form that preceded the accident (*n* = 287). When there was a more credible date on the same form (e.g., the date of first treatment, date of first assessment, signature date) it was chosen. Finally, the first date of healthcare consultation and the associated provider type was retrieved from either the healthcare provider form or the healthcare billing database. If the healthcare provider form and the healthcare billing database specified that the patient saw different types of providers on the same day, the provider listed on the form was chosen.

### Independent variables

The independent variables were grouped in terms of *predisposing*, *enabling* and *needs* characteristics in accordance with the Andersen behavioral model of health services [44, 45]. We decided to account for the particular importance of work-related factors by adding the workplace as a subcategory of predisposing factors.

### Predisposing characteristics

#### Demographic

Age and sex were obtained from the claim file.

#### Social

The preferred language was available from both the employer and the worker forms. The French and English categories were combined for the bivariable and multivariable analyses because their association with the dependent variable did not significantly differ.

#### Workplace

The job tenure was obtained from the claim file. Union membership was available from both the worker and the employer forms. The employers indicated on their form if they doubted that the injury was work-related. This variable was used as a proxy for an adversarial reaction from the employer. The national occupational code (NOC) contained in the claim file was used to determine the physical demands of the job (manual, non-manual and mixed work) using an exposure matrix [46, 47]. The sector of economic activity [48] was obtained from the claim file. The business size was determined according to the number of employees included in the claim file for the years 2004 and 2005; these numbers were averaged, and the results were used to dichotomize the employers into those with 20 employees or fewer and those with more than 20 employees. In Ontario, businesses with more than 20 employees have re-employment obligations, while smaller businesses do not [49]. The employers also specified on their form whether they had an early return to work program.

### Enabling resources

#### Financial

The worker’s weekly gross income from the claim file was multiplied by 52 to obtain the annual gross income.

#### Organizational

The community size and an urban/rural indicator were determined by applying the postal code conversion file [50] to the postal code from the claim file.

### Needs

Both the worker and the employer reported on their form if, to their knowledge, the employee had had a similar injury in the past. The WSIB claim file revealed whether the worker had filed any previous lost time claim in Ontario (Canada). The healthcare provider form contained information about task limitations and the abilities to use public transportation and to operate a motor vehicle. The claim file contained information about the nature of the injury and the part of the body affected. The Canadian Standard Association’s Z795 classification [51] for part of body and nature of injury was used by the WSIB coders, who applied it approximately 6 months after the date of injury using all information that was available at that time. We categorized the affected body part in four anatomical regions (Additional file 1: Table S1). We dichotomized the nature of the injury into less-severe cases (non-specific backache) and more-severe case (disc disorders, sciatica, herniated lumbar disc, radiculitis; Additional file 1: Table S1) based on a previously used classification [52, 53].

### Health behavior

The number of days between the accident date and the first health care consultation was calculated. The healthcare billing database was used to identify the different types of healthcare providers who billed for services within the first 4 weeks after the accident.

### Compensation process

The dates of claim registration and approval were obtained from the claim file, and the number of days between the accident and the claim registration and approval was calculated.

### Analysis

We generated frequencies (categorical variables) or means and standard deviations (continuous variables) for all variables. Bivariable analyses were conducted between all the workers’ characteristics and the type of first healthcare provider using ANOVA (post hoc tests: Bonferroni correction or Games-Howell (unequal variances) [54]) and Pearson’s chi-square tests. All comparisons were 2-tailed and were considered statistically significant at *P* < 0.05.

Missing data levels were low (<5 %) for all variables included in the study with the exception of job tenure, sector of economic activity, employer doubt that the injury was work related, restricted use of public transportation or a motor vehicle, any task limitations, and the availability of an early return to work program. Globally, 3.5 % of the values were missing. Our analysis of the missing value patterns led us to assume that the data were missing at random [55]. Consequently, imputation of missing values was performed with multiple imputations using the Markov Chain Monte Carlo simulation. All the available information was used, with the exception of the sector of economic activity and the community size, to respect the 100-parameter limit for multiple imputations in SPSS. A total of 20 imputed databases were created [56]. Pooled estimates were obtained using Rubin algorithms [57].

Three multiple logistic regression models were developed to identify the most significant predictors of the type of first healthcare provider from among the workers’ characteristics. The approach used to build each model was the same [58]. All the independent variables (except health behaviors after the first consultation and compensation process indicators) with a *P* < 0.25 in the bivariate analyses were entered into a multivariable logistic regression model. To create the preliminary model, the least significant variables were removed from the model one by one until all the variables had a *P* < 0.25. We then tried to reintroduce all of the variables that were excluded or were not initially included one by one. The final model was built by reintroducing variables into the model if they had a *P* < 0.25 or if their introduction altered at least one of the other variables’ pooled coefficients by more than 20 %. Linearity in the logit was assessed graphically for continuous predictors. Multicollinearity was investigated using the variance inflation factor. The goodness-of-fit of the final model was assessed using the area under the receiver operating characteristic curve (ROC) [59]. Areas under the ROC ≥0.90–1.00 were considered excellent, ≥0.80–90 considered good, ≥0.70–80 considered fair, ≥0.60–0.70 considered poor and ≥0.50–60 fail. We report the adjusted odds ratios (OR) with 95 % confidence intervals for each independent variable of the final model. We performed all analyses using SPSS for Mac (version 22.0, IBM Corporation, Armonk, NY, USA).

## Results

### Description

Of the 5520 compensated workers who were analyzed, the majority first saw a medical doctor (*n* = 4710; 85.3 %); 11.4 % (*n* = 627) first saw a chiropractor, 3.2 % (*n* = 174) first saw a physiotherapist, and 0.2 % (*n* = 9) first saw a nurse practitioner. Because the number of workers who first sought care from a nurse practitioner was very small, we excluded those workers from our bivariate and multivariable analyses. The characteristics of the analyzed sample are presented in Table 1. The analyzed sample was predominantly male (61.9 %) with an average age of 36.6 years, and manufacturing was the most frequently represented sector of activity (24.0 %). Those characteristics are similar to those of the overall population of workers compensated by the WSIB [11].

**Table 1:** Descriptive statistics of the compensated workers’ characteristics (*n* = 5520)

Individual characteristics		
	*n* or mean	% or SD
Predisposing characteristics		
*Demographic*:		
Sex; *n* (%)		
Male	3417	61.9 %
Female	2103	38.1 %
Age; mean (SD)	36.6	10.9
*Social*:		
Language; *n* (%)		
English	5123	92.8 %
French	89	1.6 %
Other	247	4.5 %
Missing	61	1.1 %
*Workplace*:		
Job tenure (years); mean (SD) (1571 missing)	7.8	8.1
Union member; *n* (%)		
Yes	2819	51.1 %
No	2625	47.6 %
Missing	76	1.4 %
Employer doubts the accident is work-related; *n* (%)		
Yes	592	10.7 %
No	4193	76.0 %
Missing	735	13.3 %
Physical demands; *n* (%)		
Manual	3538	64.1 %
Non-manual	655	11.9 %
Mixed-manual	1158	21.0 %
Missing	169	3.1 %
Sector of economic activity; *n* (%)		
Agriculture-related service	41	0.7 %
Fishing/trapping	1	0.0 %
Logging and forestry	7	0.1 %
Mining, quarrying and oil wells	15	0.3 %
Manufacturing	1324	24.0 %
Construction	278	5.0 %
Transportation and storage	324	5.9 %
Communication and other utilities	85	1.5 %
Wholesale trade	335	6.1 %
Retail trade	664	12.0 %
Real estate operator or insurance agent	28	0.5 %
Business service	189	3.4 %
Government service	47	0.9 %
Educational service	30	0.5 %
Health and social service	717	13.0 %
Accommodation, food and beverage service	228	4.1 %
Other service	160	2.9 %
Missing	1047	19.0 %
Early return to work program; *n* (%)		
Yes	4437	80.4 %
No	704	12.8 %
Missing	379	6.9 %
Number of employees; *n* (%)		
20 or fewer	1621	29.4 %
More than 20	3887	70.4 %
Missing	12	0.2 %
Enabling resources		
*Financial*:		
Gross personal income per year in Canadian dollars; mean (SD) (1 missing)	37581	17016
*Organizational*:		
Community size; *n* (%)		
1,500,000+	1982	35.9 %
500,000–1,499,999	696	12.6 %
100,000–499,999	1648	29.9 %
10,000–99,999	519	9.4 %
Less than 10,000	661	12.0 %
Missing	14	0.3 %
Urban/rural indicator; *n* (%)		
Rural	784	14.2 %
Urban	4716	85.4 %
Missing	20	0.4 %
Needs		
Previous similar injury; *n* (%)		
Yes	1937	35.1 %
No	3417	61.9 %
Missing	166	3.0 %
Previous 100 % wage compensation claim; *n* (%)		
Yes	2501	45.3 %
No	3019	54.7 %
Restricted use of public transportation; *n* (%)		
Yes	44	0.8 %
No	4823	87.4 %
Missing	653	11.8 %
Restricted operation of a motor vehicle; *n* (%)		
Yes	116	2.1 %
No	4779	86.6 %
Missing	625	11.3 %
Any task limitations; *n* (%)		
Yes	3674	66.6 %
No	1281	23.2 %
Missing	565	10.2 %
Nature of injury; *n* (%)		
Least severe	5227	94.8 %
Most severe	284	5.2 %
Part of body; *n* (%)		
Upper back pain	492	8.9 %
Low back pain	4528	82.2 %
Multiple regions	315	5.7 %
Back pain (unspecified upper or low)	176	3.2 %
Health behavior		
Days between the accident and the first health care consultation; mean (SD)	2.1	3.9
First healthcare provider; *n* (%)		
Chiropractor	627	11.4 %
Physician	4710	85.3 %
Physiotherapist	174	3.2 %
Nurse	9	0.2 %
Compensation process		
Days between the accident and the registration of the claim; mean (SD) (1 missing)	7.7	8.3
Days between the accident and the approval of the claim; mean (SD)	32.3	58.4

*SD* standard deviation

### Factors associated with the type of first healthcare provider

#### Bivariate results

The results of the bivariate analyses of the workers’ characteristics and the type of first healthcare provider sought are presented in Table 2. The workers who first consulted a physiotherapist were significantly older than the workers who first consulted a medical doctor (*P* = 0.002). They also had a longer time interval before the first consultation (*P* < 0.001) and were more likely to use an additional type of care than the workers who first sought chiropractic or medical care (*P* < 0.001). The workers who chose a chiropractor as their first healthcare provider had significantly more years of work experience (*P* = 0.002), a greater income (*P* < 0.001) and a longer time before claim approval (*P* < 0.001) than the workers who first sought medical care. They were also less likely to live in community larger than 1,500,000 inhabitants (*P* < 0.001) and to have a manual job (*P* = 0.041) than the workers who first consulted a physician or a physiotherapist. The workers who first consulted a medical doctor had significantly less severe injuries (*P* = 0.001), fewer previous similar injuries (*P* < 0.001) and a shorter time before registering their claim than the workers who first consulted a chiropractor or a physiotherapist (*P* < 0.001).

**Table 2:** Results of bivariate analyses of worker characteristics associated with the first health care provider sought and the compensation duration (*n* = 5511)

Variables	Association with the first healthcare provider						
	DC		MD		PT		*P*-value
	*n* or mean	% or SD	*n* or mean	% or SD	*n* or mean	% or SD	
Individual characteristics							
Predisposing characteristics							
*Demographic*:							
Sex; *n* (%)							
Male	393	62.7 %	2919	62.0 %	100	57.5 %	0.445
Female	234	37.3 %	1791	38.0 %	74	42.5 %	
Age; mean (SD)	40	11	39	11	42	10	0.002^a^
*Social*:							
Language; *n* (%)							
English or French	599	96.8 %	4436	95.3 %	168	96.6 %	0.184
Other	20	3.2 %	221	4.7 %	6	3.4 %	
*Workplace*:							
Job tenure (years); mean (SD)	9.5	8.9	7.6	8	8.8	8.6	0.002^b^
Union member; *n* (%)							
Yes	341	55.0 %	2381	51.3 %	93	53.8 %	0.193
No	279	45.0 %	2261	48.7 %	80	46.2 %	
Employer doubts the accident is work-related; *n* (%)							
Yes	69	13.2 %	505	12.3 %	16	11.0 %	0.753
No	455	86.8 %	3602	87.7 %	129	89.0 %	
Physical demands; *n* (%)							
Manual	374	60.9 %	3050	66.9 %	110	65.1 %	0.041
Non-manual	81	13.2 %	548	12.0 %	24	14.2 %	
Mixed-manual	159	25.9 %	962	21.1 %	35	20.7 %	
Sector of economic activity; *n* (%)							
Agriculture, fishing/trapping, logging and forestry, mining, quarrying and oil wells	5	1.0 %	56	1.5 %	3	2.2 %	0.503
Manufacturing	139	29.1 %	1147	29.8 %	37	27.4 %	
Construction	31	6.5 %	243	6.3 %	4	3.0 %	
Transportation and storage	35	7.3 %	280	7.3 %	7	5.2 %	
Communication and other utility	9	1.9 %	74	1.9 %	2	1.5 %	
Wholesale trade	37	7.7 %	288	7.5 %	10	7.6 %	
Retail trade	76	15.9 %	566	14.7 %	21	15.6 %	
Real estate operator or insurance agent	1	0.2 %	26	0.7 %	1	0.7 %	
Business service	18	3.8 %	163	4.2 %	8	5.9 %	
Government service	10	2.1 %	33	0.9 %	3	2.2 %	
Educational service	1	0.2 %	29	0.8 %	0	0.0 %	
Health and social service	80	16.7 %	606	15.7 %	30	22.2 %	
Accommodation, food and beverage service	19	4.0 %	205	5.3 %	4	3.0 %	
Other service	17	3.6 %	137	3.6 %	5	3.7 %	
Early return to work program; *n* (%)							
Yes	502	84.2 %	3783	86.5 %	144	88.3 %	0.235
No	94	15.8 %	590	13.5 %	19	11.7 %	
Number of employees; *n* (%)							
20 or fewer	210	33.6 %	1359	28.9 %	50	29.1 %	0.053
More than 20	415	66.4 %	3343	71.1 %	122	70.9 %	
Enabling resources							
*Financial*:							
Gross personal income per year ($CAN); mean (SD)	40054	17300	37173	16948	39732	17021	<0.001^c^
*Organizational*:							
Community size; *n* (%)							
1,500,000 +	185	29.6 %	1719	36.6 %	77	44.3 %	<0.001
500,000–1,499,999	60	9.6 %	614	13.1 %	20	11.5 %	
100,000–499,999	207	33.1 %	1395	29.7 %	44	25.3 %	
10,000–99,999	78	12.5 %	424	9.0 %	16	9.2 %	
Less than 10,000	96	15.3 %	545	11.6 %	17	9.8 %	
Urban/rural indicator; *n* (%)							
Rural	105	16.8 %	657	14.0 %	20	11.5 %	0.098
Urban	520	83.2 %	4035	86.0 %	154	88.5 %	
Needs							
Previous similar injury; *n* (%)							
Yes	288	47.1 %	1565	34.3 %	83	48.3 %	<0.001
No	323	52.9 %	2997	65.7 %	89	51.7 %	
Previous 100 % wage compensation claim; *n* (%)							
Yes	301	48.0 %	2112	44.8 %	86	49.4 %	0.179
No	326	52.0 %	2598	55.2 %	88	50.6 %	
Restricted use of public transportation; *n* (%)							
Yes	6	1.2 %	38	0.9 %	0	0.0 %	0.432
No	510	98.8 %	4165	99.1 %	142	100.0 %	
Restricted operation of a motor vehicle; *n* (%)							
Yes	8	1.6 %	106	2.5 %	2	1.4 %	0.306
No	503	98.4 %	4127	97.5 %	142	98.6 %	
Any task limitations; *n* (%)							
Yes	402	77.5 %	3160	73.8 %	107	72.8 %	0.182
No	117	22.5 %	1123	26.2 %	40	27.2 %	
Nature of injury; *n* (%)							
Least severe	581	92.7 %	4488	95.3 %	158	90.8 %	0.001
Most severe	46	7.3 %	222	4.7 %	16	9.2 %	
Part of body; *n* (%)							
Upper back pain	53	8.5 %	246	9.0 %	13	7.5 %	0.494
Low back pain	522	83.3 %	3859	81.9 %	147	84.5 %	
Multiple regions	40	6.4 %	267	5.7 %	8	4.6 %	
Back pain (unspecified upper or low)	12	1.9 %	158	3.4 %	6	3.4 %	
Health behavior							
Days between the accident and the first health care consultation; mean (SD)	2.3	3.8	2	3.7	5.1	6.4	<0.001^d^
Additional type of care sought within the first 4 weeks; *n* (%)							
Chiropractor	−	−	507	10.8 %	3	1.7 %	<0.001*
Physician	144	23.0 %	−	−	102	58.6 %	
Physiotherapist	25	4.0 %	1213	25.8 %	−	−	
No additional type of care	476	75.9 %	3026	64.2 %	70	40.2 %	
Compensation process							
Days between the accident and the registration of the claim; mean (SD)	8.9	9.7	7.5	8.1	9.1	8.3	<0.001^e^
Days between the accident and the approval of the claim; mean (SD)	43	100	31	51	37	50	<0.001^f^

*: Chi-squared for “no additional type of care”

^a:^ Workers who chose a physician as the first healthcare provider were significantly younger than workers choosing physiotherapists

^b:^ The workers who chose a physician as the first healthcare provider had significantly fewer years of experience compared with the workers who chose chiropractors

^c^: The workers who chose chiropractors as the first healthcare provider had significantly higher incomes than the workers who chose physicians

^d^: The workers who chose a physiotherapist as the first healthcare provider had a significantly higher time interval between the accident date and the first healthcare consultation compared with the workers who chose a chiropractor or physician

^e^: The workers who chose a physician as the first healthcare provider had significantly lower time intervals between the accident date and the date of claim registration compared with the workers who chose a chiropractor or physiotherapist

^f^: The workers who chose a chiropractor as the first healthcare provider had a significantly higher time interval between the accident date and the claim approval compared with the workers who chose a physician

#### Multivariable results

Our three final multivariable logistic regression models are presented in Table 3. The models were fit using the data from the pooled estimates of multiple imputations. All the ORs obtained from the listwise analysis (not reported) were within 10 % of the reported pooled ORs. All the independent variables in the final models influenced the dependent variable in the same direction as in the bivariable analyses.

**Table 3:** Variables associated with the type of first healthcare provider in the multivariable logistic regression

	DC vs MD			PT vs MD			PT vs DC		
	(*n* = 5337)			(*n* = 4721)			(*n* = 800)		
	OR	95 % CI	*P*-value	OR	95 % CI	*P*-value	OR	95 % CI	*P*-value
Predisposing characteristics									
*Demographic*:									
Sex (male)	−	−	−	0.78	(0.57 to 1.07)	0.130	0.71	(0.49 to 1.04)	0.080
Age (10 years)	0.94	(0.85 to 1.03)	0.157	1.19	(1.03 to 1.38)	0.019	1.28	(1.07 to 1.54)	0.008
*Workplace*:									
Job tenure (years)	1.02	(1.01 to 1.03)	0.004	−	−	−	0.98	(0.96 to 1.01)	0.229
Physical demands									
Manual	Reference			−	−	−	reference		
Non-manual	1.23	(0.95 to 1.61)	0.115	−	−	−	0.80	(0.46 to 1.37)	0.412
Mixed-manual	1.35	(1.10 to 1.65)	0.004	−	−	−	0.63	(0.40 to 0.99)	0.044
Early return to work program (yes)	0.77	(0.61 to 0.98)	0.035	−	−	−	−	−	−
Enabling resources									
*Financial*:									
Gross personal income per year ($10,000)	1.06	(1.01 to 1.12)	0.018	1.06	(0.97 to 1.16)	0.205	−	−	−
*Organizational*:									
Community size (inhabitants)									
1,500,000+	0.66	(0.50 to 0.86)	0.002	1.50	(0.88 to 2.55)	0.142	2.58	(1.42 to 4.67)	0.002
500,000–1,499,999	0.58	(0.41 to 0.82)	0.002	1.10	(0.57 to 2.10)	0.787	1.98	(0.95 to 4.12)	0.069
100,000–499,999	0.88	(0.67 to 1.14)	0.332	1.03	(0.63 to 1.68)	0.912	1.26	(0.68 to 2.33)	0.472
10,000–99,999	1.09	(0.78 to 1.51)	0.621	1.22	(0.70 to 2.13)	0.575	1.23	(0.58 to 2.61)	0.597
Less than 10,000	reference			reference			reference		
Needs									
Previous similar injury (yes)	1.60	(1.34 to 1.90)	<0.001	1.71	(1.25 to 2.33)	0.001	−	−	−
Any task limitations (yes)	1.18	(0.95 to 1.48)	0.135	−	−	−	−	−	−
Nature of injury (more severe)	1.39	(0.99 to 1.96)	0.054	2.03	(1.21 to 3.41)	0.010	1.49	(0.80 to 2.78)	0.205
Part of body									
Upper back pain	reference			−	−	−	−	−	−
Low back pain	1.01	(0.75 to 1.38)	0.928	−	−	−	−	−	−
Multiple regions	1.19	(0.76 to 1.86)	0.447	−	−	−	−	−	−
Back pain (unspecified upper or low)	0.60	(0.31 to 1.16)	0.127	−	−	−	−	−	−
Constant	0.12	(0.07 to 0.20)	<0.001	0.01	(0.01 to 0.02)	<0.001	0.10	(0.04 to 0.23)	<0.001
Area under the ROC curve	0.62	(0.60 to 0.65)	<0.001	0.63	(0.59 to 0.67)	<0.001	0.64	(0.60 to 0.69)	<0.001

A value greater than 1 represents increased odds of seeking care from the first type of healthcare provider in the comparison, and a value lower than 1 indicates decreased odds. For example, in the DC vs MD comparison, if the odds ratio is 1.2 for a specific category of a categorical variable, the subjects within that category have 20 % higher odds of seeking a chiropractor than a medical doctor compared with the subjects in the reference category

*CI* confidence intervals, *DC* chiropractor, *MD* physician, *OR* Odds ratio, *PT* Physiotherapist

Among the largest effects observed were the impacts of the community size and nature of injury. The odds of first seeing a chiropractor, rather than a physician or physiotherapist, were significantly lower among those who lived in communities with more than 500,000 inhabitants and particularly among those who lived in communities with more than 1,500,000 inhabitants (DC/MD *OR* = 0.66, *P* = 0.002; PT/DC *OR* = 2.58, *P* = 0.002). The odds of first seeing a physiotherapist rather than a medical doctor were significantly higher when the nature of the injury was more severe (*OR* = 2.03, *P* = 0.010).

Previously injured workers had higher odds of first consulting a chiropractor (*OR* = 1.60, *P* < 0.001) or a physiotherapist (*OR* = 1.71, *P* = 0.001) rather than a medical doctor. The odds of first seeing a chiropractor compared with a physician were significantly lower when early return to work programs were available (*OR* = 0.77, *P* = 0.035). Workers who held a mixed-manual job had significantly greater odds of first seeking chiropractic care rather than medical (DC/MD *OR* = 1.35, *P* = 0.004) or physiotherapy care (PT/DC *OR* = 0.63, *P* = 0.044). Increased age corresponded to greater odds of consulting a physiotherapist compared with a chiropractor (*OR* = 1.28, *P* = 0.008) or a physician (*OR* = 1.19, *P* = 0.019).

Longer job tenure (*OR* = 1.02, *P* = 0.004) and higher income (*OR* = 1.06, *P* = 0.018) both significantly increased the odds of first seeing a chiropractor rather than a physician, but the magnitude of the effects was relatively small, and the clinical relevance is unclear.

## Discussion

### Summary of the main findings

The workers who first sought physiotherapy care were significantly older than those who first chose chiropractic and medical care. They also had more severe injuries than the medical patients. The workers who first sought chiropractic care had significantly longer job tenures, less access to early return to work programs, and higher personal incomes than the workers who sought medical care. They were also less frequently living in communities with more than 1,500,000 inhabitants and were more likely to have mixed-manual jobs compared with the medical and physiotherapy patients. The workers who reported having had a previous similar injury tended to choose chiropractic and physiotherapy care over medical care.

It is worth noting that the workers who initially sought physiotherapy experienced longer time intervals between the accident and the first healthcare consultation and were more likely to seek additional types of care within the first month after the injury, according to the bivariate analysis. The workers who first sought medical care had their claims registered earlier, while those who first sought chiropractic care had their claims accepted later. This suggests that the type of first healthcare provider might influence the claim administration process.

### Comparisons with other studies

According to previous studies, older patients more often choose medical care (with or without physiotherapy) over chiropractic care [12, 18, 19, 27, 28]. However, the average difference in mean age was relatively small (3 years or less), and the clinical significance of the difference is not clear. Our results are slightly different because there was no significant difference in age between the chiropractic and medical care patients, but the physiotherapy patients were slightly older. Our sample of compensated workers included more men than women, but we did not find significant differences between the sexes in the type of care sought. Findings from studies conducted in the general population provided divergent results, as some did not find significant differences in the type of care sought [17, 19], while others suggested that men were more likely to seek chiropractic care [12, 18, 28, 30, 31].

Among the workplace factors we investigated, union membership, employers’ doubts about the work-relatedness of the injury and the number of employees were not associated with the type of care sought. We found that workers with a longer job tenure had a greater likelihood of seeking chiropractic care over medical care. A possible explanation might be that workers wait to have a stronger employment link before seeking a complementary and alternative healthcare provider for an occupational injury. We also found that the availability of an early return to work program was associated with higher odds of seeking medical care over chiropractic care only when controlling for other predisposing characteristics. It is counterintuitive to think that an early return to work program could influence the initial type of care sought; instead, this association might be explained by other related factors. It is reasonable to hypothesize that workplaces with early return to work programs are more concerned with employee health and might also have policies or organizational factors that facilitate access to medical care, such as onsite medical appointments or flexible working hours. Compared with the manual workers, the mixed-manual workers had greater odds of seeking a chiropractor than a physician or a physiotherapist. It has been previously demonstrated that the type of occupation could influence the type of care sought [37]. Compared with workers in service occupations, workers in skilled or semi-skilled occupations were more likely to be treated by a chiropractor rather than a medical physician [37]. An American study also revealed that workers whose employer selected the initial healthcare provider were much less likely to consult a chiropractor alone or in combination with a physician [37]. Even when workers refer themselves to the provider of their choice, as in the Ontarian context, chiropractors with more employer references received significantly more workers’ compensation patients [60].

In Ontario, the WSIB will cover the healthcare of the workers suffering from an occupational injury. However, some chiropractors and physiotherapists (physicians bill directly to the Ontario health insurance plan) might not bill directly the WSIB, meaning that the patients have to pay at the point of service and then submit a claim for coverage to the WSIB. This might explain why higher income was associated with greater odds of seeking chiropractic care over medical care, even though the average income difference was relatively small. Previous studies in different contexts also found that patients with lower incomes had a greater tendency to seek medical care [13, 24], while patients with higher incomes were more likely to seek physiotherapy care [12, 23] or chiropractic care [16, 31].

Our results suggest that workers from large urban communities have greater odds of seeking medical and physiotherapy care over chiropractic care than workers from smaller communities. A study conducted in Saskatchewan concluded the opposite, with fewer chiropractic patients than medical patients living in rural areas [13]. The differences in care-seeking between the two provinces might be explained by the regional supply of healthcare providers and the timing of the data collection. Canadians who consult chiropractors only are more likely to lack access to a regular family physician than patients who see other combinations of providers [16]. An American study found that a greater supply of chiropractors in an area increased the number of chiropractic consultations and decreased the number of primary care physician visits for back pain [61]. Another possible reason for the differences found between Ontario and Saskatchewan is the demographic disparities between the two provinces, since Saskatchewan only has two major urban centres (Regina and Saskatoon), both of which have less than 250,000 inhabitants. Consequently, the largest communities in Saskatchewan are categorized in the third largest category of communities in Ontario (100,000–499,000 inhabitants).

We found that workers who reported a previous similar injury were more likely to seek physiotherapy and chiropractic care, while those who had previously received income compensation did not vary in the type of care they sought. It is reasonable to think that workers will seek care that they perceived as effective for a similar condition, compensated or not, in the past. Previous studies have found that back pain patients are more likely to seek the type of care they previously sought [62], and this association was particularly strong for chiropractic care [20, 62]. Our results suggest that workers suffering from more severe conditions are more likely to seek physiotherapy (*OR* = 2.03; *P* = 0.010) and chiropractic care (*OR* = 1.36; *P* =0.054) than medical care. The nature of the injury (more or less severe) was partly informed by the content of the healthcare provider form. It is therefore possible that the observed difference is attributable to differential reporting by the different healthcare provider types. Another plausible explanation is that workers who seek chiropractic and physiotherapy care are more likely to report a previous similar injury, and they might also be more likely to return directly to chiropractic or physiotherapy care if they were referred to those types of care in the past [21–23, 32]. It is therefore possible that in the Ontarian context of workers compensation for back pain, patients with more severe conditions are more likely to seek a physiotherapist or a chiropractor. This finding is contrary to those of previous studies, which reported that patients with more severe pain, disability, comorbidity and a lower general health status are more likely to see a physician than a chiropractor [13, 17, 18, 23–26, 30, 33]. We only retrieved two studies with conclusions that were consistent with our findings: a Swedish study that suggested that chiropractic patients are more affected by pain than primary care patients [36] and an Australian study that suggested that chiropractic patients have more co-morbidities and depression than medical patients do [35]. Because the severity of the injury was determined using nature of injury codes, it is possible that the workers with “less severe” injuries also experienced high levels of pain and suffered from many comorbidities. We used the task limitations reported by the healthcare provider as a proxy for functional limitations, and we did not find significant variations between the different types of providers. Given that this variable was dichotomized, it could be hiding more subtle differences. The region (s) of the spine affected did not seem to influence the type of care sought in our sample.

The time interval between the accident and the first healthcare consultation was longer for physiotherapy patients. However, this time interval (mean: 5.2 days; median: 3.0 days) was considerably shorter than when a physician referral was required (median: 16 days) [63], suggesting that temporal access to physiotherapy care has been improved by policy changes, albeit not as quickly as medical and chiropractic care have been affected. This is important because more rapid access to physiotherapy care was previously associated with a shorter duration of financial compensation [62, 64]. The medical patients experienced faster claim registrations and approvals, while the chiropractic patients had a longer time interval before their claims were approved. Whether the timing and quality of the information that the healthcare providers included on their forms or the type of provider itself influenced the claim approval should be investigated. It is unclear if the time to claim approval does impact the duration of financial compensation, as suggested by a Californian study [53] because the possibility of immortal time bias was not considered. The time interval between the accident and the first healthcare consultation could be considered as immortal, since return to work could not occur before the consultation. Therefore, an incorrect consideration of this unexposed time period in the design or analysis could lead to immortal time bias and artificially increase the magnitude of the association between the timing of the consultation and the compensation duration. It is unclear which factors influence the timing of claim approvals, but the fact that chiropractic care is considered complementary and alternative care might be worth investigating because the differences in registration time were relatively small between the three types of providers. Another finding of interest is the higher use of other healthcare professionals among physiotherapy patients. Previous studies suggested that early aggressive use of health resources might have an iatrogenic effect [33, 62, 65, 66]. Physiotherapy care has been previously associated with lower use of radiographic investigation [17] and higher medication use [62] compared with medical care. Chiropractic care was associated with lower use of medication, radiographic investigation, and surgery [17, 62]. Among the different types of initial care, including medical care and physiotherapy, chiropractic care was considered more “guideline coherent” for low back pain among an American self-insured workforce [62]. A possible explanation for the high use of medical consultations among the workers who initially sought physiotherapy care is that at the time of our study, Ontarian physiotherapists could not order radiographic imaging or prescribe medication. Additionally, direct access to physiotherapy for occupational injury is a relatively recent phenomenon in Ontario (January 2004), and it is possible that physiotherapists and/or workers are not familiar with their new role in the workers’ compensation process.

Overall, our findings are consistent with those of previous studies. They highlight the impact of work-related factors and organizational enabling factors (community size) on the type of care sought for occupational back pain. Contrary to previous studies, we found that workers who first sought a physician had lower odds of having a severe injury compared with those who first sought a physiotherapist.

According to Andersen, access to care to care is considered equitable when demographic and needs factors primarily account for the use of healthcare resources and inequitable when social and enabling resources are important contributors [67]. In this study, the largest effects were observed among the needs (severity of injury) and enabling factors (community size). To increase the equitability of access, enabling factors might be modulated to attenuate their impact on the type of care sought.

### Strengths and limitations

The strengths of this study include a large sample that provided sufficient statistical power for multivariable modeling. From the data that the WSIB routinely collects from workers, employers and healthcare providers, we were able to retrieve a large number of predisposing, enabling and need variables. To limit misclassification, we considered two sources of information (billing data and the healthcare provider form) to construct our main dependent variable. Our use of multiple imputations enabled us to adequately address missing values in our multivariable models.

The forms used by the WSIB during the compensation process were designed for administrative purposes, and their psychometrics proprieties have not been measured. This may be of particular interest regarding the need variables because our results differ from those of previous studies. The rationale behind our classification of the severity of injury was associated with the duration of previous financial compensation [52, 53], suggesting appropriate construct validity, but we do not know how this classification compares to the established measures of pain and functional status. In our sample, the nature of the injury was recorded by the WSIB coders approximately 6 months after the injury using all of the information available from the claim. The use of an independent coder may have made the coding more objective, but the healthcare providers provide some of that information, and it is possible that the information that was provided differed systematically according to the types of healthcare providers. The same rationale could apply to the evaluation of task restrictions. Although we have no evidence of differential misclassification, this is a possibility we cannot completely rule out.

Our multivariable models only included variables that were available through the WSIB. It is likely that some relevant variables were not included in our analysis, and thus, residual confounding is possible. The results of our multivariable regression models have limited scope in terms of predicting the type of first healthcare provider (area under the ROC curve between 0.62 and 0.64). The addition of potentially relevant variables would have provided the opportunity to study interesting associations and might have improved the predictive power of our models. It would have been interesting to include formal education in our analysis because previous findings suggest that higher levels of formal education increase the probability of seeing a chiropractor [16, 30, 31] or a physiotherapist in addition to a physician [12, 32]. Wider socio-demographic information such as marital status and ethnicity could as well play a role in the choice of a healthcare provider. The regional supply of healthcare providers might also have influenced the type of provider consulted [61]. The presence/absence of co-morbidities and chronic musculoskeletal conditions were previously identified as relevant determinants [13]. Finally, the worker’s health beliefs and expectations were omitted from our analysis, and they might play an important role in the choice of a healthcare provider [26, 68] and the return to work process [69].

Our results originate from a large Ontarian cohort of back pain patients who received financial compensation from the WSIB and should be representative of the population under study. However, we excluded a significant proportion of the workers randomly selected because they had missing or aberrant data regarding our main dependent variable. It is possible that the excluded subjects significantly differ from the one we analyzed by an unknown factor and therefore limit the representativeness of the analyzed sample. We analyzed data collected one year after the policy change that enabled workers to directly seek physiotherapy care. It is possible that our analysis capture early adopters of the new policy and that the characteristics of the workers first consulting a physiotherapist now differ from the ones assessed in 2005. Generalization to other provinces or conditions should be performed with caution. The type of occupational care sought may vary from one province to another [60]. In Ontario, medical care is delivered through a publically funded healthcare system that is free at the point of service; while chiropractic and physiotherapy care are covered by the WSIB with a fee schedule that is lower than would be charged to a non-WSIB patient. Therefore, the observed associations might differ in other healthcare and compensation systems.

### Recommendations for future research

Most of the studies that investigated care-seeking patterns for occupational injuries or back pain used cross-sectional designs or performed secondary data analyses of insurer administrative databases. Our results suggest that administrative data poorly predict initial care-seeking patterns; therefore, qualitative research would be better able to identify the main factors that influence the type of care that injured workers seek and to understand the mechanism underlying the initial choice of a healthcare provider.

## Conclusions

Ontarian workers who received compensation for occupational back pain mainly seek medical doctors; some visit chiropractors, and a few see physiotherapists as their first healthcare provider. The type of first healthcare provider sought for occupational back pain is influenced by injury-and work-related factors as well as the worker’s age, income and community size. Contrary to previous studies, the workers who first seek a physician do not have higher odds of having a severe injury.

## Additional file

Additional file 1: Table S1. Part of Body and nature of injuries codes [51] used for claim selection. (DOCX 15 kb)

## Abbreviations

ANOVA: Analysis of variance
CI: Confidence interval
CIHR: Canadian institutes for health research
DC: Doctor of chiropractic
IWH: Institute for work and health
MD: Medical doctor
NOC: National occupational code
OR: Odds ratio
PT: Physiotherapist
ROC: Receiver operating characteristics
R-RTW: Readiness for return to work
SD: Standard deviation
WSIB: Workplace safety and insurance board
