# Supplementary material for: Workers’ characteristics associated with the type of healthcare provider first seen for occupational back pain
Source: BMC Musculoskelet Disord. 2016 Oct 18;17:428. doi: 10.1186/s12891-016-1298-y (PMC5069865; doi:10.1186/s12891-016-1298-y)
Supplement: Additional file 1: Table S1. — Part of Body and nature of injuries codes [51] used for claim selection. (DOCX 15 kb) [file 12891_2016_1298_MOESM1_ESM.docx]

## Additional file 1: Table S1. Part of Body and nature of injuries codes[50] used for claim selection

| **Part of body** | |
| --- | --- |
| **Upper back pain** | Thoracic region, unspecified (23200); Cervico-thoracic region (23201); Thoracic region, n.e.c. (23290). |
| **Low back pain** | Lumbar region of the back (23100); Dorso-lumbar region (23202); Sacral region, unspecified (23300); Lumbo-sacral region (23301); Sacral region (23390); Coccyx (23400); Back, low, unspecified location (23901). |
| **Multiple regions** | Multiple back regions (23800). |
| **Back pain, unspecified location** | Spine, spinal cord, unspecified (23000); Back, including spine, spinal cord (23900). |
| **Nature of injury** | |
| **Least severe cases** | Sprains, strains, tears, unspecified (2100); Bruises, contusions (4300); Sprains and bruises (8200); Back pain, hurt back (9720); Non-specific injuries and disorders (9790); Lumbago (17220); Dorsopathies (17290); Facet syndrome (17292); Inflammation & irritation of joints, tendons, muscles and connective tissues (17390); Spasms or tremors (41210). |
| **More severe cases** | Sciatica (17210); Disc disorders, unspecified (17230); Herniated disc (17231); Disc disorders (17239); Radiculitis (17293). |
|  |  |
